# Molecular Mechanisms in Mood Regulation Involving the Circadian Clock

**DOI:** 10.3389/fneur.2017.00030

**Published:** 2017-02-07

**Authors:** Urs Albrecht

**Affiliations:** ^1^Department of Biology, Unit of Biochemistry, University of Fribourg, Fribourg, Switzerland

**Keywords:** clock genes, depression, monoamines, glucocorticoids, neurogenesis

## Abstract

The circadian system coordinates activities and functions in cells and tissues in order to optimize body functions in anticipation to daily changes in the environment. Disruption of the circadian system, due to irregular lifestyle such as rotating shift work, frequent travel across time-zones, or chronic stress, is correlated with several diseases such as obesity, cancer, and neurological disorders. Molecular mechanisms linking the circadian clock with neurological functions have been uncovered suggesting that disruption of the clock may be critically involved in the development of mood disorders. In this mini-review, I will summarize molecular mechanisms in which clock components play a central role for mood regulation. Such mechanisms have been identified in the monoaminergic system, the HPA axis, and neurogenesis.

A plethora of human genetic studies have identified polymorphisms in clock genes that associate with psychiatric disorders [reviewed in Ref. (1)]. This suggested that abnormalities in clock genes may be one of the causes for the development of mood disorders. At the cellular level, clock genes (*Bmal1, Clock, Per, Cry, Rev-erb*, and *Ror*) make up an autoregulatory transcriptional/translational feedback loop with a period of about 24 h (Figure 1, top gray circle) [reviewed in Ref. (2)]. These clock genes and their proteins not only self-promote their own temporally fluctuating transcription but they also regulate transcription of target genes (Figure 1) and/or modulate key molecular pathways *via* protein–protein interactions, such as the monoaminergic system, the HPA axis, or neurogenic pathways.

**Figure 1:**
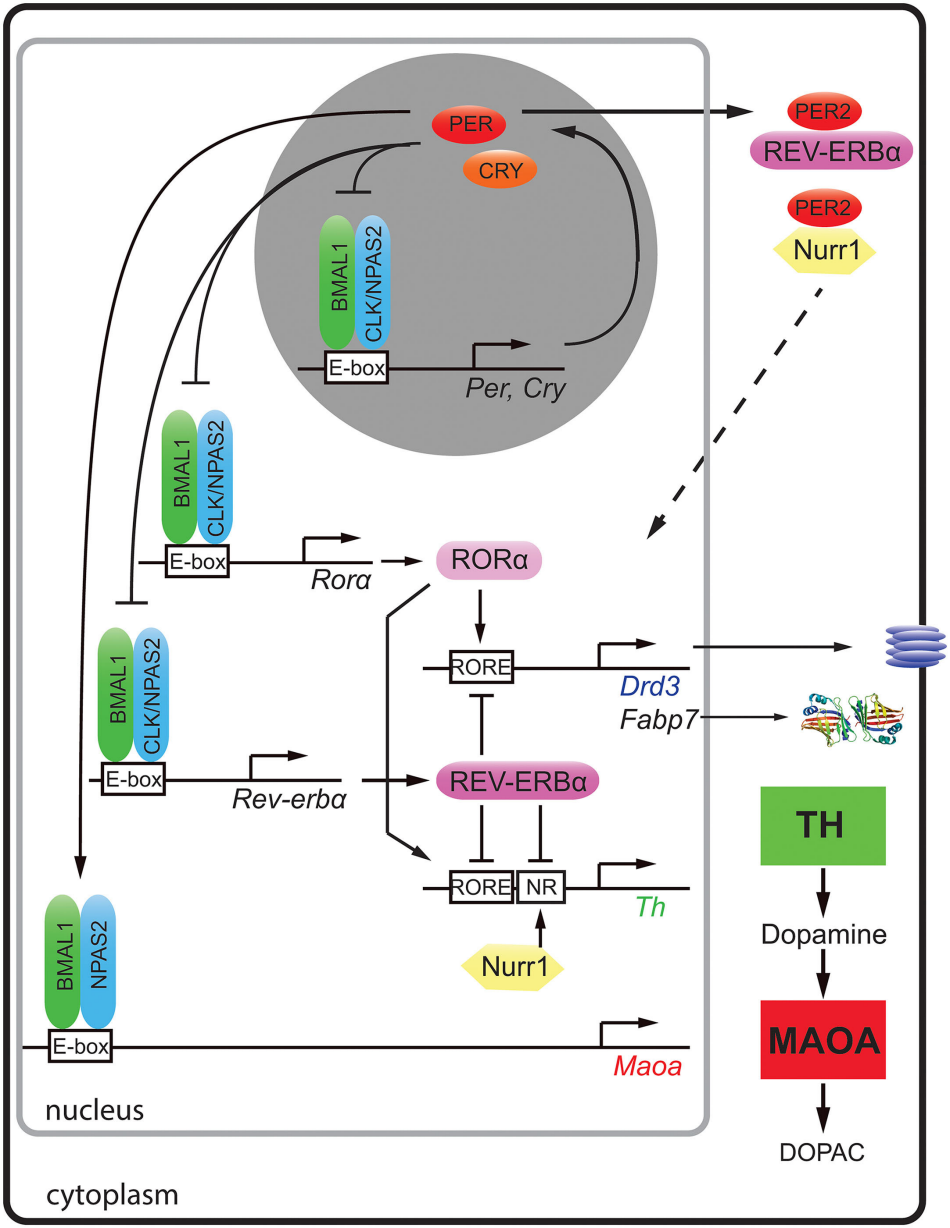
**Molecular regulation of clock and clock-controlled genes of the monoaminergic system and neurogenesis**. The clock proteins BMAL1 (green), CLOCK (blue), and NPAS2 (blue) bind to E-box elements present in the promoters of clock genes (*Per, Cry, Ror*α, and *Rev-erb*α) and the clock-controlled gene for monoamine oxidase A (*Maoa*). PER (red) and cryptochrome (CRY, orange) proteins inhibit the action of BMAL1/CLOCK and BMAL1/NPAS2 heterodimers, respectively. The nuclear receptors [retinoic orphan receptor α (RORα, rose)] and REV-ERBα (purple) both bind to RORE elements of dopamine receptor 3 (*Drd3*), fatty acid binding protein 7 (*Fabp7*), and tyrosine hydroxylase (*Th*) in a competitive manner and activate or inhibit their expression, respectively. The nuclear receptor Nurr1 (yellow) regulates *Th via* its NR promoter element. *Via* protein–protein interactions, PER2 can modulate the actions of REV-ERBα and Nurr1 (hatched arrow). This regulation results in temporally regulated expression of the dopamine synthesizing (TH, green square) and degrading enzymes (MAOA, red square) leading to fluctuating levels of dopamine in the striatum.

## Transcriptional Regulation of Monoamine Signaling by Clock Components

Neuroimaging studies in humans indicated that the monoaminergic system (dopamine, serotonin, and noradrenaline) was altered in subjects with mood disorders (3). This was further supported by optogenetic studies, in which control of neuronal activity of dopamine neurons in mice modulated mood, anxiety, and reward, confirming the importance of the monoaminergic system in mood-related behaviors (4, 5).

Interestingly, several studies described daily changes in dopamine, serotonin, and noradrenaline levels [reviewed in Ref. (6)]. Because these molecules modulate arousal, motivation, and reward, one would expect them to be targeted at the activity period of the day in order to avoid conflicts with sleep signals. Hence, monoaminergic signaling is likely to be regulated by the circadian clock, either directly or indirectly. In the last years, several investigations aimed at uncovering the role of circadian clock components in the direct transcriptional regulation of elements important for monoaminergic signaling, such as the enzymes monoamine oxidase (MAO) and tyrosine hydroxylase (TH) both key enzymes for the degradation and synthesis of dopamine, respectively.

Dopamine degradation is under clock control. This was first suggested by the observation that the clock components BMAL1 and NPAS2 transcriptionally activated a luciferase reporter driven by the murine monoamine oxidase A (*Maoa*) promoter in a circadian fashion. This indicated that these two clock components directly regulated *Maoa* transcription (Figure 1). This notion was further strengthened by the observation that BMAL1 protein was recruited to the *Maoa* promoter in brain tissue (7). Interestingly, the regulation by BMAL1/NPAS2 was modulated by PER2 in a positive fashion, but not in the predicted negative manner (Figure 1). This lead to increased *Maoa* mRNA levels (7). This finding suggested potential tissue specific regulatory factors that turned PER2 into a positive regulator of BMAL1/NPAS2-driven transcriptional regulation in the striatum. As a consequence of lack of PER2, not only *Maoa* mRNA but also MAOA protein levels were decreased. Hence, dopamine degradation was reduced, and dopamine levels in the nucleus accumbens were increased. This was paralleled by a depression-resistant-like phenotype and changes in neuronal activity in response to MAO inhibitors in mice (7). These findings strongly suggested that the degradation of monoamines was clock modulated. It is very likely that the described clock-mediated regulation of monoamines is relevant for humans, because single-nucleotide polymorphisms in *Per2, Bmal1*, and *Npas2* associated in an additive fashion with seasonal affective disorder or winter depression (8).

A recent study showed that not only dopamine degradation but also dopamine synthesis is under clock influence. The mouse, rat, and human *Th* promoters were repressed by REV-ERBα, and they were activated by retinoic orphan receptor α (RORα) and nuclear receptor-related protein 1 (NURR1) (9). Chromatin immunoprecipitation experiments revealed that REV-ERBα and NURR1 were binding to the *Th* promoter in an antagonistic manner (9). In accordance with this mechanism (Figure 1), *Rev-erb*α knock-out mice displayed elevated *Th* mRNA and protein levels leading to increased dopamine amounts and firing rate in the striatum (9, 10). As a consequence, these animals showed less depression-like and anxiety-like behavior compared to wild-type animals (9). The temporal regulation of TH may be further modulated through protein–protein interactions. For example, PER2 has the potential to interact with both REV-ERBα and NURR1 proteins (11), which would allow temporal synchronization of the action of these two nuclear receptors (Figure 1, top right, hatched arrow). This is, however, a speculation and needs verification.

Interestingly, REV-ERBα and RORα were described to regulate the expression of the dopamine D3 receptor gene (*Drd3*) in an antagonistic manner (12) (Figure 1). This provided a molecular explanation why this receptor was expressed in a diurnal manner in the striatum (13). DRD3 inhibits adenylyl cyclase through inhibitory G-proteins [reviewed in Ref. (14)] and mutation of DRD3 in mice suggested an involvement of this receptor in mediating emotional behavior and depression in mice (15). A role of NPAS2 in the regulation of *Drd3* has also been suggested (16), although it is unclear how NPAS2 would regulate the *Drd3* promoter. Taken together, it appears that REV-ERBα and RORα synchronize dopamine production and the expression of DRD3 in the striatum probably to optimally restrict dopamine signaling in the striatum to a particular time window. This implies that the targeting of DRD3 and/or REV-ERBα/RORα by pharmacological agents may benefit from timed application. This would reduce dosage and diminish side effects such as weight gain, which is observed often in patients treated for mood disorders.

## Molecular Regulation of Components of the HPA Axis by Clock Proteins

Epidemiological studies suggested that stressful life events play a role in the etiology of depression (17), and hypercortisolemia was observed in a subset of patients with depression [reviewed in Ref. (18)]. Furthermore, antidepressant treatment appeared to stabilize the function of the HPA axis *via* the serotonergic system (19), suggesting an involvement of the HPA axis and glucocorticoids in mood regulation [reviewed in Ref. (20)].

**Figure 2:**
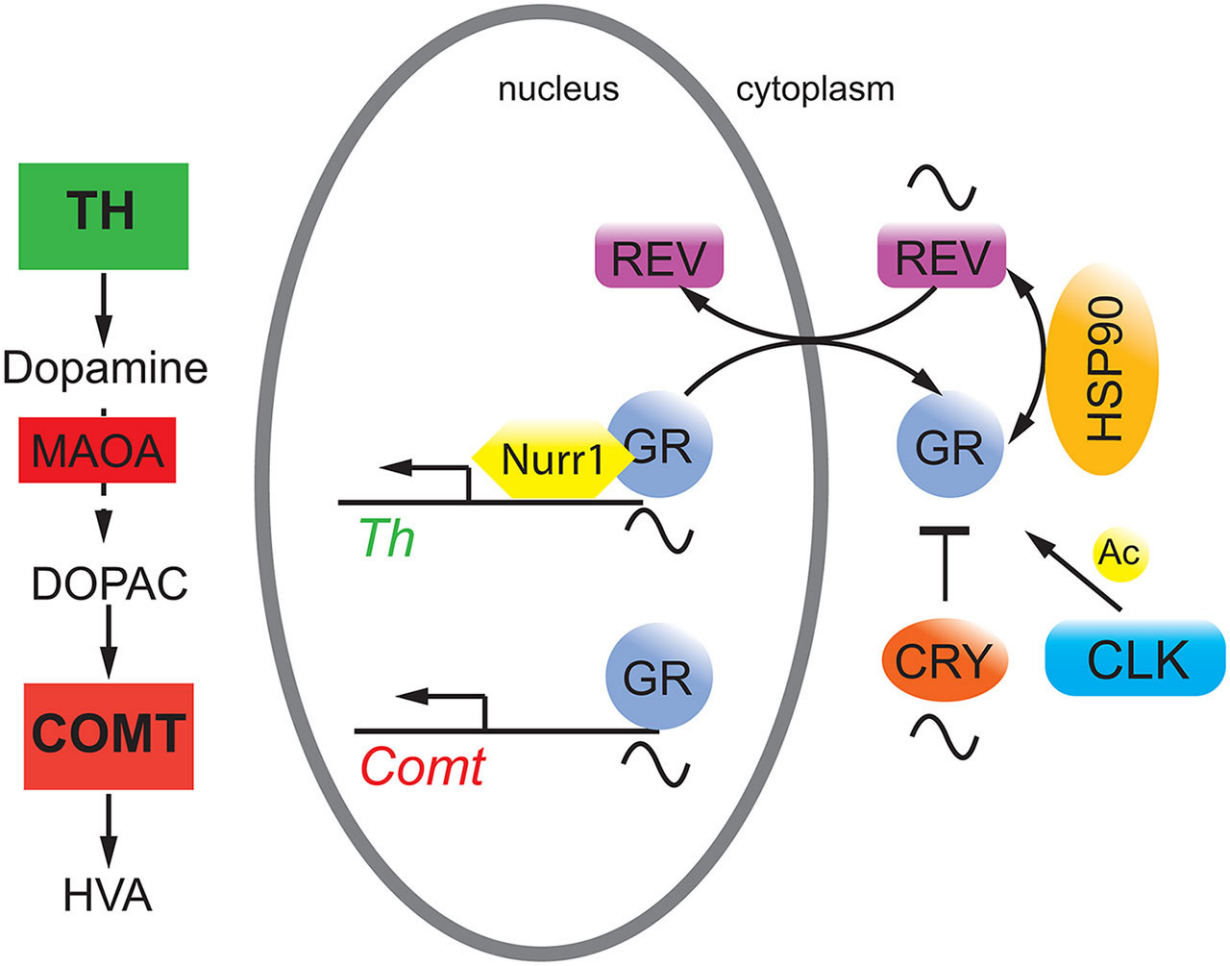
**Hypothetical model on the interaction of circadian clock proteins with the glucocorticoid receptor (GR)**. REV-ERBα (REV, purple) gates nuclear localization of the GR (gray) *via* an unknown mechanism probably involving heat shock protein 90 (HSP90, yellow). GR function is inhibited by cryptochrome (CRY, orange) proteins and is modulated by CLOCK (blue) *via* acetylation (Ac), although it is unclear whether this happens in the cytoplasm and/or the nucleus. GR regulates target genes such as *catechol-O-methyltransferase* (*Comt*) whose protein is an enzyme (COMT, red square) that degrades 3,4-dihydroxyphenylacetic acid (DOPAC) to homovanillic acid (HVA). GR may also interact with Nurr1 to modulate *tyrosine hydroxylase* (*Th*) expression thereby influencing dopamine production.

Conditional mutagenesis in mice of the glucocorticoid receptor (GR) in the nervous system provided evidence for the importance of GR signaling in emotional behavior (21). Overexpression of GR lead to depressive-like behavior, and these mice showed enhanced sensitization to cocaine (22), consistent with observations that GR may be a potential target to reduce cocaine abuse (23). Interestingly, GR bound to NURR1 thereby increasing the transcriptional potential of NURR1 to induce TH (24) (Figure 1). Hence, the amount of nuclear GR appeared to be important for this function. Although glucocorticoids displayed circadian rhythmicity [reviewed in Ref. (25)], GR expression was constant over 24 h in the liver, which applies most likely to the brain as well. However, GR nuclear localization appeared to be gated by REV-ERBα in the liver with nuclear GR levels high at zeitgeber time 20 (activity period of mice) (26). If this would apply to the brain, REV-ERBα would gate binding of GR to NURR1 for induction of the *Th* promoter (Figure 2). As illustrated above, mood-related behavior and dopamine levels were changed in *Rev-erb*α*^−/−^* mice, and this may also involve GR, which regulates catechol-*O*-methyltransferase (26), an enzyme degrading the MAOA product 3,4-dihydroxyphenylacetic acid to homovanillic acid. Therefore, it is likely that the monoaminergic system and the glucocorticoid pathway are linked *via* GR.

The cryptochrome (CRY) proteins interact with GR in a ligand-dependent manner in mouse liver leading to rhythmic repression of GR activity (27). Additionally, the CRY proteins participate in glucocorticoid-dependent suppression of the HPA axis and the production of endogenous glucocorticoids (27). Mice lacking *Cry1* showed depression-like behavior combined with reduced levels of dopamine in the striatum (28). This phenotype was most likely the result of the effects of CRY on both pathways illustrated in Figures 1 and 2. Furthermore, GR was acetylated by CLOCK, which lead to decreased sensitivity to glucocorticoids in the morning in humans and to an increased sensitivity at night when acetylation was reversed (29).

Recently, CHRONO, a protein that acts as a repressor in the circadian clock mechanism similar to CRY2 appeared to have the potential to interact with GR as well (30). Interestingly, *Chrono* mRNA was induced in the hypothalamus after stress stimulation whereas *Cry2* mRNA was not. This suggested that CHRONO may be a stress-inducible repressor of the circadian clock coupling the clock with the HPA axis (30). However, it is not known whether *Chrono* knock-out mice display alterations in mood-related behaviors.

## Transcriptional Regulation of Neurogenesis by Clock Proteins

Adult neurogenesis is an important process to replace lost or dysfunctional neurons with new neurons produced from neuronal stem cells. Most of them are found in the subventricular zone lining the lateral ventricles and the subgranular zone of the hippocampal dentate gyrus. Environmental stimuli, such as stress, physical activity, sleep deprivation, enriched living conditions, and jet-lag, can influence adult hippocampal neurogenesis in mammals (31–35). These environmental stimuli directly affect the circadian clock as well [reviewed in Ref. (36)], suggesting that the clock plays a mediator role between environmental change and neurogenesis. Animal studies showed that chronic stress and depression-inducing behavior reduced hippocampal neurogenesis while antidepressants enhanced it (37), suggesting a connection between neurogenesis and depressive behavior (38). Hence, change of the clock by environmental stimuli may affect neurogenesis, which in turn affects mood-related behaviors. Interestingly, neurogenesis varied over the day (39–42), and mutations in clock genes affected adult hippocampal neurogenesis (28, 43–46). The effect of the clock on this process was at least in part due to the control of the timing of cell-cycle entry and exit of quiescent neural progenitor cells (QNPs) (47). For example, absence of *Per2* abolished the gating of cell-cycle entrance of QNPs (43, 47), whereas lack of *Bmal1* resulted in constitutively high levels of proliferation and delayed cell-cycle exit (46, 47).

On the molecular level evidence of direct clock gene-mediated regulation of neurogenesis is scarce. The mechanism of *Clock*- and *Bmal1*-mediated neuronal differentiation appeared to be associated with the neurogenic transcription factor NeuroD1 (48), although a direct regulation of its promoter by clock genes was not shown. In contrast, the regulation of fatty acid binding protein 7 (*Fabp7*), also termed brain lipid-binding protein, by the clock component REV-ERBα has been elucidated (44). FABP7 facilitates the solubility of long-chain fatty acids and is implicated in cell growth and differentiation (49). It affects neuronal differentiation (50) and is a marker for neuronal progenitor cells (51, 52). The promoter of the *Fabp7* gene was directly suppressed by REV-ERBα, and this suppression was relieved by RORα, a positive competitor of REV-ERBα (Figure 1) (44). Mice lacking *Rev-erb*α displayed increased levels of FABP7, which was associated with alterations in mood-related behaviors, changes in hippocampus-dependent cognitive performance, and increased hippocampal neurogenesis (44).

Taken together, this overview illustrates multiple levels of molecular mood regulation with REV-ERBα (and PER2 as REV-ERBα modulator) being involved in all of the processes described; regulation of the monoaminergic system, the HPA axis, and neurogenesis.

In the future, a better understanding of the hypothetical molecular processes illustrated in Figure 2 will be of great importance, because it is unknown whether CRY and CLOCK affect GR function in the nucleus or the cytoplasm. This would distinguish whether the influence of these two clock components is directly on transcription or on modulation of GR protein stability and transport, which would influence GR-mediated transcription in an indirect manner. Furthermore, the posttranslational regulation of REV-ERBα is poorly understood with the exception of its residues S55/S59, which are phosphorylated by GSK3β and may mediate cellular sensitivity to lithium (53). Time–of-day-dependent phosphorylation sites on REV-ERBα and GR (54) may contribute to the gated regulation of nuclear presence of these two receptors and hence on the regulation of metabolism and mood-related behaviors.

## Author Contributions

UA wrote the manuscript and prepared the figures.

## Conflict of Interest Statement

The author declares that the research was conducted in the absence of any commercial or financial relationships that could be construed as a potential conflict of interest.

## References

[1] Bellivier F Geoffroy PA Etain B Scott J. Sleep- and circadian rhythm-associated pathways as therapeutic targets in bipolar disorder. Expert Opin Ther Targets (2015) 19:747–63. 10.1517/14728222.2015.1018822 25726988

[2] Takahashi JS. Molecular components of the circadian clock in mammals. Diabetes Obes Metab (2015) 17(Suppl 1):6–11. 10.1111/dom.12514 26332962 PMC4560116

[3] Meyer JH. Applying neuroimaging ligands to study major depressive disorder. Semin Nucl Med (2008) 38:287–304. 10.1053/j.semnuclmed.2008.02.007 18514084

[4] Chaudhury D Walsh JJ Friedman AK Juarez B Ku SM Koo JW Rapid regulation of depression-related behaviours by control of midbrain dopamine neurons. Nature (2013) 493:532–6. 10.1038/nature11713 23235832 PMC3554860

[5] Tye KM Mirzabekov JJ Warden MR Ferenczi EA Tsai HC Finkelstein J Dopamine neurons modulate neural encoding and expression of depression-related behaviour. Nature (2013) 493:537–41. 10.1038/nature11740 23235822 PMC4160519

[6] McClung CA. Circadian genes, rhythms and the biology of mood disorders. Pharmacol Ther (2007) 114:222–32. 10.1016/j.pharmthera.2007.02.003 17395264 PMC1925042

[7] Hampp G Ripperger JA Houben T Schmutz I Blex C Perreau-Lenz S Regulation of monoamine oxidase A by circadian-clock components implies clock influence on mood. Curr Biol (2008) 18:678–83. 10.1016/j.cub.2008.04.012 18439826

[8] Partonen T Treutlein J Alpman A Frank J Johansson C Depner M Three circadian clock genes Per2, Arntl, and Npas2 contribute to winter depression. Ann Med (2007) 39:229–38. 10.1080/07853890701278795 17457720

[9] Chung S Lee EJ Yun S Choe HK Park SB Son HJ Impact of circadian nuclear receptor REV-ERBalpha on midbrain dopamine production and mood regulation. Cell (2014) 157:858–68. 10.1016/j.cell.2014.03.039 24813609

[10] Jager J O’Brien WT Manlove J Krizman EN Fang B Gerhart-Hines Z Behavioral changes and dopaminergic dysregulation in mice lacking the nuclear receptor Rev-erbalpha. Mol Endocrinol (2014) 28:490–8. 10.1210/me.2013-1351 24552589 PMC3968406

[11] Schmutz I Ripperger JA Baeriswyl-Aebischer S Albrecht U. The mammalian clock component PERIOD2 coordinates circadian output by interaction with nuclear receptors. Genes Dev (2010) 24:345–57. 10.1101/gad.564110 20159955 PMC2816734

[12] Ikeda E Matsunaga N Kakimoto K Hamamura K Hayashi A Koyanagi S Molecular mechanism regulating 24-hour rhythm of dopamine D3 receptor expression in mouse ventral striatum. Mol Pharmacol (2013) 83:959–67. 10.1124/mol.112.083535 23429911

[13] Akhisaroglu M Kurtuncu M Manev H Uz T. Diurnal rhythms in quinpirole-induced locomotor behaviors and striatal D2/D3 receptor levels in mice. Pharmacol Biochem Behav (2005) 80:371–7. 10.1016/j.pbb.2004.11.016 15740778

[14] Civelli O Bunzow JR Grandy DK Molecular diversity of the dopamine receptors. Annu Rev Pharmacol Toxicol (1993) 33:281–307. 10.1146/annurev.pharmtox.33.1.281 8494342

[15] Xu M Koeltzow TE Santiago GT Moratalla R Cooper DC Hu XT Dopamine D3 receptor mutant mice exhibit increased behavioral sensitivity to concurrent stimulation of D1 and D2 receptors. Neuron (1997) 19:837–48. 10.1016/S0896-6273(00)80965-4 9354330

[16] Ozburn AR Falcon E Twaddle A Nugent AL Gillman AG Spencer SM Direct regulation of diurnal Drd3 expression and cocaine reward by NPAS2. Biol Psychiatry (2015) 77:425–33. 10.1016/j.biopsych.2014.07.030 25444159 PMC4315729

[17] Kendler KS Kessler RC Walters EE MacLean C Neale MC Heath AC Stressful life events, genetic liability, and onset of an episode of major depression in women. Am J Psychiatry (1995) 152:833–42. 10.1176/ajp.152.6.833 7755111

[18] Wolkowitz OM Burke H Epel ES Reus VI. Glucocorticoids. mood, memory, and mechanisms. Ann N Y Acad Sci (2009) 1179:19–40. 10.1111/j.1749-6632.2009.04980.x 19906230

[19] Carvalho LA Garner BA Dew T Fazakerley H Pariante CM. Antidepressants, but not antipsychotics, modulate GR function in human whole blood: an insight into molecular mechanisms. Eur Neuropsychopharmacol (2010) 20:379–87. 10.1016/j.euroneuro.2010.02.006 20231081 PMC2982744

[20] Massart R Mongeau R Lanfumey L. Beyond the monoaminergic hypothesis: neuroplasticity and epigenetic changes in a transgenic mouse model of depression. Philos Trans R Soc Lond B Biol Sci (2012) 367:2485–94. 10.1098/rstb.2012.0212 22826347 PMC3405682

[21] Tronche F Kellendonk C Kretz O Gass P Anlag K Orban PC Disruption of the glucocorticoid receptor gene in the nervous system results in reduced anxiety. Nat Genet (1999) 23:99–103. 10.1038/12703 10471508

[22] Wei Q Lu XY Liu L Schafer G Shieh KR Burke S Glucocorticoid receptor overexpression in forebrain: a mouse model of increased emotional lability. Proc Natl Acad Sci U S A (2004) 101:11851–6. 10.1073/pnas.0402208101 15280545 PMC511063

[23] Deroche-Gamonet V Sillaber I Aouizerate B Izawa R Jaber M Ghozland S The glucocorticoid receptor as a potential target to reduce cocaine abuse. J Neurosci (2003) 23:4785–90. 12805318 10.1523/JNEUROSCI.23-11-04785.2003 PMC6740779

[24] Carpentier R Sacchetti P Segard P Staels B Lefebvre P. The glucocorticoid receptor is a co-regulator of the orphan nuclear receptor Nurr1. J Neurochem (2008) 104:777–89. 10.1111/j.1471-4159.2007.05055.x 17986226

[25] Oster H Challet E Ott V Arvat E de Kloet ER Dijk DJ The functional and clinical significance of the 24-h rhythm of circulating glucocorticoids. Endocr Rev (2016):er20151080. 10.1210/er.2015-1080 27749086 PMC5563520

[26] Okabe T Chavan R Fonseca Costa SS Brenna A Ripperger JA Albrecht U REV-ERBalpha influences the stability and nuclear localization of the glucocorticoid receptor. J Cell Sci (2016) 129:4143–54. 10.1242/jcs.190959 27686098 PMC5117207

[27] Lamia KA Papp SJ Yu RT Barish GD Uhlenhaut NH Jonker JW Cryptochromes mediate rhythmic repression of the glucocorticoid receptor. Nature (2011) 480:552–6. 10.1038/nature10700 22170608 PMC3245818

[28] Schnell A Sandrelli F Ranc V Ripperger JA Brai E Alberi L Mice lacking circadian clock components display different mood-related behaviors and do not respond uniformly to chronic lithium treatment. Chronobiol Int (2015) 32:1075–89. 10.3109/07420528.2015.1062024 26317159

[29] Charmandari E Chrousos GP Lambrou GI Pavlaki A Koide H Ng SS Peripheral CLOCK regulates target-tissue glucocorticoid receptor transcriptional activity in a circadian fashion in man. PLoS One (2011) 6:e25612. 10.1371/journal.pone.0025612 21980503 PMC3182238

[30] Goriki A Hatanaka F Myung J Kim JK Yoritaka T Tanoue S A novel protein, CHRONO, functions as a core component of the mammalian circadian clock. PLoS Biol (2014) 12:e1001839. 10.1371/journal.pbio.1001839 24736997 PMC3988004

[31] Gould E McEwen BS Tanapat P Galea LA Fuchs E. Neurogenesis in the dentate gyrus of the adult tree shrew is regulated by psychosocial stress and NMDA receptor activation. J Neurosci (1997) 17:2492–8. 9065509 10.1523/JNEUROSCI.17-07-02492.1997 PMC6573503

[32] van Praag H Kempermann G Gage FH. Running increases cell proliferation and neurogenesis in the adult mouse dentate gyrus. Nat Neurosci (1999) 2:266–70. 10.1038/6368 10195220

[33] Guzman-Marin R Suntsova N Stewart DR Gong H Szymusiak R McGinty D. Sleep deprivation reduces proliferation of cells in the dentate gyrus of the hippocampus in rats. J Physiol (2003) 549:563–71. 10.1113/jphysiol.2003.041665 12679377 PMC2342950

[34] Kempermann G Kuhn HG Gage FH. More hippocampal neurons in adult mice living in an enriched environment. Nature (1997) 386:493–5. 10.1038/386493a0 9087407

[35] Gibson EM Wang C Tjho S Khattar N Kriegsfeld LJ. Experimental ‘jet lag’ inhibits adult neurogenesis and produces long-term cognitive deficits in female hamsters. PLoS One (2010) 5:e15267. 10.1371/journal.pone.0015267 21152025 PMC2995744

[36] Golombek DA Rosenstein RE. Physiology of circadian entrainment. Physiol Rev (2010) 90:1063–102. 10.1152/physrev.00009.2009 20664079

[37] Eisch AJ Petrik D. Depression and hippocampal neurogenesis: a road to remission? Science (2012) 338:72–5. 10.1126/science.1222941 23042885 PMC3756889

[38] Snyder JS Soumier A Brewer M Pickel J Cameron HA. Adult hippocampal neurogenesis buffers stress responses and depressive behaviour. Nature (2011) 476:458–61. 10.1038/nature10287 21814201 PMC3162077

[39] Goergen EM Bagay LA Rehm K Benton JL Beltz BS. Circadian control of neurogenesis. J Neurobiol (2002) 53:90–5. 10.1002/neu.10095 12360586

[40] Kochman LJ Weber ET Fornal CA Jacobs BL. Circadian variation in mouse hippocampal cell proliferation. Neurosci Lett (2006) 406:256–9. 10.1016/j.neulet.2006.07.058 16930842

[41] Tamai S Sanada K Fukada Y. Time-of-day-dependent enhancement of adult neurogenesis in the hippocampus. PLoS One (2008) 3:e3835. 10.1371/journal.pone.0003835 19048107 PMC2585014

[42] Gilhooley MJ Pinnock SB Herbert J. Rhythmic expression of per1 in the dentate gyrus is suppressed by corticosterone: implications for neurogenesis. Neurosci Lett (2011) 489:177–81. 10.1016/j.neulet.2010.12.011 21163331

[43] Borgs L Beukelaers P Vandenbosch R Nguyen L Moonen G Maquet P Period 2 regulates neural stem/progenitor cell proliferation in the adult hippocampus. BMC Neurosci (2009) 10:30. 10.1186/1471-2202-10-30 19327139 PMC2714160

[44] Schnell A Chappuis S Schmutz I Brai E Ripperger JA Schaad O The nuclear receptor REV-ERBalpha regulates Fabp7 and modulates adult hippocampal neurogenesis. PLoS One (2014) 9:e99883 10.1371/journal.pone.0099883 24932636 PMC4059695

[45] Malik A Kondratov RV Jamasbi RJ Geusz ME. Circadian clock genes are essential for normal adult neurogenesis, differentiation, and fate determination. PLoS One (2015) 10:e0139655. 10.1371/journal.pone.0139655 26439128 PMC4595423

[46] Ali AA Schwarz-Herzke B Stahr A Prozorovski T Aktas O von Gall C. Premature aging of the hippocampal neurogenic niche in adult Bmal1-deficient mice. Aging (Albany NY) (2015) 7:435–49. 10.18632/aging.100764 26142744 PMC4505169

[47] Bouchard-Cannon P Mendoza-Viveros L Yuen A Kaern M Cheng HY. The circadian molecular clock regulates adult hippocampal neurogenesis by controlling the timing of cell-cycle entry and exit. Cell Rep (2013) 5:961–73. 10.1016/j.celrep.2013.10.037 24268780

[48] Kimiwada T Sakurai M Ohashi H Aoki S Tominaga T Wada K. Clock genes regulate neurogenic transcription factors, including NeuroD1, and the neuronal differentiation of adult neural stem/progenitor cells. Neurochem Int (2009) 54:277–85. 10.1016/j.neuint.2008.12.005 19121353

[49] Feng L Hatten ME Heintz N. Brain lipid-binding protein (BLBP): a novel signaling system in the developing mammalian CNS. Neuron (1994) 12:895–908. 10.1016/0896-6273(94)90341-7 8161459

[50] De Rosa A Pellegatta S Rossi M Tunici P Magnoni L Speranza MC A radial glia gene marker, fatty acid binding protein 7 (FABP7), is involved in proliferation and invasion of glioblastoma cells. PLoS One (2012) 7:e52113. 10.1371/journal.pone.0052113 23284888 PMC3528762

[51] Young JK Heinbockel T Gondre-Lewis MC. Astrocyte fatty acid binding protein-7 is a marker for neurogenic niches in the rat hippocampus. Hippocampus (2013) 23:1476–83. 10.1002/hipo.22200 23996503 PMC3859315

[52] Giachino C Basak O Lugert S Knuckles P Obernier K Fiorelli R Molecular diversity subdivides the adult forebrain neural stem cell population. Stem Cells (2014) 32:70–84. 10.1002/stem.1520 23964022 PMC4259462

[53] Yin L Wang J Klein PS Lazar MA. Nuclear receptor Rev-erbalpha is a critical lithium-sensitive component of the circadian clock. Science (2006) 311:1002–5. 10.1126/science.1121613 16484495

[54] Robles MS Humphrey SJ Mann M Phosphorylation is a central mechanism for circadian control of metabolism and physiology. Cell Metab (2016) 25(1):118–27. 10.1016/j.cmet.2016.10.004 27818261

